# Quantitative first-pass MRI measures increased myocardial perfusion after vasodilation in mice

**DOI:** 10.1186/1532-429X-14-S1-P55

**Published:** 2012-02-01

**Authors:** Patrick Antkowiak, Christopher M Kramer, Craig H Meyer, Brent A French, Frederick H Epstein

**Affiliations:** 1 University of Virginia, Charlottesville, VA, USA

## Summary

We used first-pass contrast-enhanced MRI to quantatively measure the myocardial Ktrans, a parameter indicating myocardial perfusion and vascular permeability, in mice with or without vasodilation. We measured a significant increase in myocardial Ktrans with vasodilation. We believe this may be the first report showing that first-pass imaging can quantify increased myocardial perfusion in mice relative to baseline.

## Background

First-pass contrast-enhanced MRI is a well-established technique for quantifying myocardial perfusion in humans and large animals and has recently emerged as a viable tool for quantifying myocardial perfusion in mice [1-3]. Applied in mice, first-pass MRI could be used to assess the roles of individual genes in perfusion and vascular permeability. The purpose of this study was test the hypothesis that first-pass contrast-enhanced MRI can measure increased myocardial perfusion after administration of a vasodilator in mice.

## Methods

Imaging was performed on a 7T Clinscan MR system equipped with a gradient system having a full strength of 650mT/m and a slew rate of 6666 mT/m/ms, and using a 30mm diameter birdcage RF coil. A saturation-recovery spiral sequence was employed, with TE = 0.36 ms, TR = 3.9ms, interleaves = 14, FOV = 25.6 x 25.6mm, matrix = 128x128, saturation delay = 40 ms, alpha = 20°, and slice thickness = 1mm. Data acquisition required 55 ms/image, approximately 40% of the murine R-R interval, and was placed in the latter part of the cardiac cycle. C57Bl6/J mice were imaged with (n=5) and without (n=5) an intraperitoneal bolus injection of the vasodilator ATL313 (Adenosine Therapeutics, Charlottesville, VA). First-pass images were acquired for one mid-ventricular short-axis slice. A dual-bolus gadolinium injection technique was used, acquiring the arterial input and tissue functions (AIF and TF) in separate scans. Myocardial Ktrans, the product of myocardial perfusion and the first-pass extraction fraction of gadolinium, was quantified using a standard Kety model deconvolution method.

## Results

**Figure 1:**
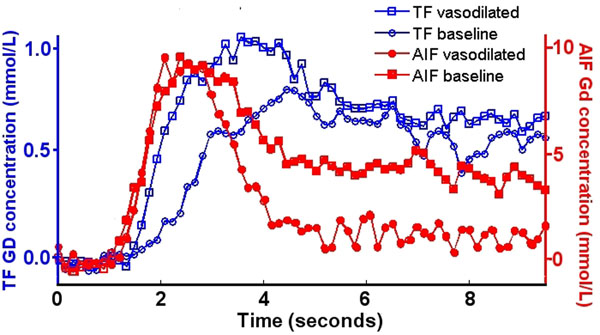
Example Gd concentration vs. time curves. The tissue function (left axis, blue) is shown for a mouse with (open blue squares) and without (open blue circles) vasodilation with ATL313. The arterial input function (right axis, red) is shown for a mouse with (closed red circles) and without (closed red squares) ATL vasodilation.

Administration of ATL313 significantly increased the heart rate in all mice. First-pass images displayed uniform tissue enhancement. Example Gd concentration vs. time curves for the TF and AIF are shown in Figure 1, comparing mice with and without ATL313 vasodilation. Myocardial Ktrans was significantly increased (p < .04) after ATL313 vasodilation (6.9 ± 2.7 ml/g/min) relative to baseline (3.3 ± 1.0 ml/g/min).

## Conclusions

These findings indicate that first-pass MRI in mice can quantitatively measure increased myocardial Ktrans with a vasodilator. Taken together with our previous studies quantifying perfusion after myocardial infarction1, these results indicate that first-pass imaging can accurately measure myocardial perfusion in mice in a variety of flow conditions.

## Funding

Funding was provided by NIH R01 EB 001763.
